# Supplementary material for: Thai psychiatrists and burnout: A national survey
Source: PLoS One. 2020 Apr 21;15(4):e0230204. doi: 10.1371/journal.pone.0230204 (PMC7173626; doi:10.1371/journal.pone.0230204)
Supplement: S1 Appendix — (DOCX) [file pone.0230204.s001.docx]

**แบบสอบถามงานวิจัย จิตแพทย์ไทยกับความเหนื่อยล้าจากการทำงาน**

**ส่วนที่ 1 แบบสอบถามข้อมูลทั่วไปและข้อมูลการทำงาน**

1. อายุ

(กรุณาเลือกระหว่าง 22-70 ปี)

1. เพศ

🞎 หญิง 🞎 ชาย

1. สถานะปัจจุบัน

🞎 จิตแพทย์ทั่วไป 🞎 จิตแพทย์เด็กและวัยรุ่น 🞎 แพทย์ประจำบ้าน

1. ระยะเวลาที่ทำงานดูแลผู้ป่วยจิตเวช(จำนวนปี) ….. ปี

(กรุณาเลือกระหว่าง 1-70 ปี)

1. สถานภาพสมรส

🞎 โสด 🞎 สมรส 🞎 หม้าย/หย่าร้าง

1. จำนวนบุตร

🞎 ไม่มี 🞎 1 คน 🞎 >1 คน

1. สถานที่ทำงาน(เลือกได้>1ข้อ)

🞎 โรงเรียนแพทย์ 🞎 โรงพยาบาลจิตเวช

🞎 โรงพยาบาลศูนย์ 🞎 โรงพยาบาลจังหวัด/โรงพยาบาลทั่วไป

🞎 โรงพยาบาลชุมชน 🞎 โรงพยาบาล/คลินิกเอกชน

1. ท่านทำงานในเขตบริการสุขภาพที่

(กรุณาเลือกระหว่างเขตที่ 1-13 หรือ 14 กรณีไม่เกี่ยวข้อง หรือ 15 กรณีไม่ทราบ)

1. จำนวนคนไข้ตรวจต่อวันโดยประมาณ(ราย)

(กรุณาเติมตัวเลข)

1. ชั่วโมงการทำงานต่อสัปดาห์

🞎<40 🞎40-50 🞎>50

1. จำนวนเวรต่อเดือน(วัน) (รวมเวรจิตเวชและเวรอื่นๆทั้งหมด)

(กรุณาเลือกระหว่าง 1-31)

1. ท่านพอใจกับรายได้ในปัจจุบันหรือไม่

กรุณาให้คะแนน 0-10 โดย 0 คือน้อยที่สุด และ 10 คือมากที่สุด ………………..

1. ท่านรู้สึกพึงพอใจกับงานที่ทำอยู่หรือไม่

กรุณาให้คะแนน 0-10 โดย 0 คือน้อยที่สุด และ 10 คือมากที่สุด ………………..

1. จำนวนวันหยุดต่อเดือน

🞎 0-2 🞎 3-5 🞎 6-8 🞎 8-10 🞎 >10

1. มีผู้ป่วยในความดูแลฆ่าตัวตายสำเร็จ

🞎ไม่มี 🞎ใน 1 เดือนที่ผ่านมา

🞎มากกว่า 1 เดือนแต่น้อยกว่า 1 ปี 🞎มากกว่า 1 ปี

1. ท่านได้รับการสนับสนุนจากข้อต่อไปนี้อย่างไร
   1. หัวหน้าหน่วยงานจิตเวช 🞎 ดี 🞎 ไม่ดี 🞎ประเมินไม่ได้
   2. หน่วยงานบริหารของรพ. 🞎 ดี 🞎 ไม่ดี 🞎ประเมินไม่ได้
   3. เพื่อนจิตแพทย์ 🞎 ดี 🞎 ไม่ดี 🞎ประเมินไม่ได้
   4. เพื่อนร่วมงานอื่นๆ 🞎 ดี 🞎 ไม่ดี 🞎ประเมินไม่ได้
   5. แพทย์แผนกอื่น 🞎 ดี 🞎 ไม่ดี 🞎ประเมินไม่ได้
   6. ครอบครัว 🞎 ดี 🞎 ไม่ดี 🞎ประเมินไม่ได้
2. เมื่อเผชิญกับความเครียด ท่านมักปรึกษาหรือปรับทุกข์กับใคร(เลือกได้มากกว่า1ข้อ)

🞎ตนเอง 🞎ผู้บังคับบัญชา 🞎เพื่อน 🞎คู่สมรส/คู่รัก/แฟน

🞎ครอบครัว 🞎Social media 🞎อื่นๆ (ระบุ) ……………………………

**ส่วนที่ 2 แบบวัดภาวะความเหนื่อยล้าในการทำงาน Maslach Burnout Inventory (MBI) ฉบับภาษาไทย**

**โปรดทำเครื่องหมายลงในช่องที่ตรงกับที่ท่านประมาณการเกิดความรู้สึกของท่าน ดังต่อไปนี้**

| เกี่ยวกับความรู้สึกของท่าน | ทุกๆวัน | สัปดาห์ละ 2-3 ครั้ง | สัปดาห์ละ 1 ครั้ง | เดือนละ 2-3 ครั้ง | เดือนละ 1 ครั้ง | ปีละ 2-3 ครั้ง | ไม่เคยรู้สึกเช่นนั้น |
| --- | --- | --- | --- | --- | --- | --- | --- |
| 1. ฉันรู้สึกจิตใจห่อเหี่ยวจากการทำงาน |  |  |  |  |  |  |  |
| 1. ฉันรู้สึกหมดแรงเมื่อสิ้นสุดเวลาทำงาน |  |  |  |  |  |  |  |
| 1. ฉันรู้สึกอ่อนเพลียเมื่อตื่นนอนตอนเช้าและรู้ว่าต้องเผชิญกับการทำงานอีกวัน |  |  |  |  |  |  |  |
| 1. ฉันสามารถเข้าถึงความรู้สึกของผู้ป่วยที่มีต่อสิ่งต่างๆได้ง่าย |  |  |  |  |  |  |  |
| 1. ฉันรู้สึกได้ว่าปฏิบัติต่อผู้ป่วยบางคนเสมือนเขาเป็นสิ่งที่ไร้ชีวิตจิตใจ |  |  |  |  |  |  |  |
| 1. การทำงานเกี่ยวกับการบริการผู้คนตลอดวันเป็นสิ่งที่ตึงเครียดสำหรับฉัน |  |  |  |  |  |  |  |
| 1. ฉันสามารถแก้ปัญหาของผู้ป่วยได้อย่างมีประสิทธิภาพ |  |  |  |  |  |  |  |
| 1. ฉันรู้สึกเหนื่อยล้าในการทำงานในภาระงาน |  |  |  |  |  |  |  |
| 1. ฉันรู้สึกว่างานของฉันมีอิทธิพลต่อชีวิตของผู้อื่น |  |  |  |  |  |  |  |
| 1. ฉันเปลี่ยนเป็นคนหยาบกระด้างและไร้เมตตาต่อผู้อื่นมากขึ้นตั้งแต่ทำงานนี้ |  |  |  |  |  |  |  |
| 1. ฉันวิตกกังวลว่างานกำลังทำให้จิตใจของฉันแข็งกระด้างขึ้น |  |  |  |  |  |  |  |
| 1. ฉันรู้สึกเปี่ยมไปด้วยพลัง |  |  |  |  |  |  |  |
| 1. ฉันรู้สึกคับข้องใจในการทำงาน |  |  |  |  |  |  |  |
| 1. ฉันรู้สึกว่ากำลังทำงานที่หนักมากเกินไป |  |  |  |  |  |  |  |
| 1. ฉันรู้สึกว่าตัวเองไม่ใส่ใจว่าอะไรจะเกิดขึ้นกับผู้ป่วยบางคน |  |  |  |  |  |  |  |
| 1. การดูแลผู้ป่วยโดยตรงทำให้ฉันรู้สึกเครียดมากเกินไป |  |  |  |  |  |  |  |
| 1. ฉันสามารถสร้างบรรยากาศที่ผ่อนคลายในการดูแลผู้ป่วยได้ง่าย |  |  |  |  |  |  |  |
| 1. ฉันรู้สึกเป็นสุขใจภายหลังการดูแลผู้ป่วย |  |  |  |  |  |  |  |
| 1. ฉันได้สร้างสรรค์สิ่งที่มีคุณค่ามากมายในการปฏิบัติงาน |  |  |  |  |  |  |  |
| 1. ฉันรู้สึกสิ้นหวัง |  |  |  |  |  |  |  |
| 1. ในการทำงานฉันสามารถเผชิญปัญหาทางอารมณ์ได้อย่างสงบ |  |  |  |  |  |  |  |
| 1. ฉันรู้สึกว่าได้รับการตำหนิจากผู้ป่วยในปัญหาบางอย่างที่เกิดขึ้น |  |  |  |  |  |  |  |

**ส่วนที่ 3 แบบวัดกลวิธีการเผชิญความเครียดเชิงรุก (The Proactive Coping Inventory: PCI)**

แต่ละข้อคำถามมี 4 ตัวเลือก ดังต่อไปนี้

(1) ไม่จริง หมายถึง ไม่เคยคิดพฤติกรรมดังกล่าวเลย

(2) จริงบางครั้ง หมายถึง เคยคิดหรือมีพฤติกรรมดังกล่าวบ้างนานๆครั้ง

(3) ค่อนข้างจริง หมายถึง เคยคิดหรือมีพฤติกรรมดังกล่าวเกิดขึ้นบางครั้ง

(4) จริงมาก หมายถึง เคยคิดหรือมีพฤติกรรมดังกล่าวอยู่เสมอ

คำชี้แจง กรุณาวงกลมหมายเลขในตัวเลือกระหว่าง 1 - 4 เพียง 1 ตัวเลือกเท่านั้น ที่ตรงกับท่านมากที่สุดในแต่ละข้อคำถาม

รวมทั้งหมด 26 ข้อคำถาม

| 1 | การตั้งเป้าหมายและการจัดการเชิงรุก | ไม่จริง(1) | จริงบางครั้ง(2) | ค่อนข้างจริง(3) | จริงมาก(4) |
| --- | --- | --- | --- | --- | --- |
| 1.1 | ฉันเป็นคนรับผิดชอบ |  |  |  |  |
| 1.2 | ฉันพยายามปล่อยให้สิ่งต่างๆเป็นไปตามทางของมัน |  |  |  |  |
| 1.3 | หลังจากบรรลุเป้าหมายหนึ่ง ฉันมองหาเป้าหมายอื่นที่ท้าทายขึ้นกว่าเดิม |  |  |  |  |
| 1.4 | ฉันชอบความท้าทายและพยายามเอาชนะเรื่องยาก ๆ |  |  |  |  |
| 1.5 | ฉันมีความใฝ่ฝันและพยายามทำความฝันนั้นให้เป็นจริง |  |  |  |  |
| 1.6 | แม้ว่าจะพบกับอุปสรรคมากมาย ฉันมักประสบความสำเร็จในการได้รับสิ่งที่ฉันต้องการ |  |  |  |  |
| 1.7 | ฉันพยายามที่จะกำหนดเป้าหมายอย่างชัดเจนกับสิ่งที่ฉันต้องการจะประสบความสำเร็จ |  |  |  |  |
| 1.8 | ฉันมักพยายามหาหนทางที่จะแก้ไขอุปสรรคไม่มีอะไรหยุดยั้งฉันได้ |  |  |  |  |
| 1.9 | ฉันมักจะเห็นภาพตนเองล้มเหลว ดังนั้นฉันจึงไม่กล้าคาดหวังสูงจนเกินไป |  |  |  |  |
| 1.10 | เวลาฉันสมัครเรียน/สมัครงาน ฉันจินตนาการว่าฉันจะได้รับคัดเลือก |  |  |  |  |
| 1.11 | ฉันเปลี่ยนอุปสรรคต่าง ๆ ให้เป็นประสบการณ์เชิงบวก |  |  |  |  |
| 1.12 | ถ้ามีคนบอกฉันว่า “ฉันไม่สามารถทำได้ ”ฉันจะทำให้เห็นว่า “ฉันสามารถทำได้” |  |  |  |  |
| 1.13 | เวลาฉันประสบปัญหา ฉันใช้ความคิดริเริ่มในการแก้ไขปัญหานั้น |  |  |  |  |
| 1.14 | เวลาฉันมีปัญหา ฉันมักมองเห็นตัวเองตกอยู่ในสถานการณ์ที่ไม่มีทางสู้ |  |  |  |  |

| 2 | การวางแผนอย่างมีกลยุทธ์ | ไม่จริง(1) | จริงบางครั้ง(2) | ค่อนข้างจริง(3) | จริงมาก(4) |
| --- | --- | --- | --- | --- | --- |
| 2.1 | ฉันมักหาวิธีที่จะแยกส่วนปัญหาที่ยุ่งยากออกเป็นองค์ประกอบย่อย ๆ เพื่อที่ฉันจะจัดการได้ง่ายขึ้น |  |  |  |  |
| 2.2 | ฉันวางแผนและทำตามแผน |  |  |  |  |
| 2.3 | ฉันแยกส่วนปัญหาออกเป็นส่วนประกอบเล็ก ๆ แล้วจัดการทีละส่วนในแต่ละครั้ง |  |  |  |  |
| 2.4 | ฉันจัดรายการความสำคัญและพยายามจดจ่อกับสิ่งที่สำคัญที่สุดก่อนเป็นลำดับแรก |  |  |  |  |

| 3 | การแสวงหาการสนับสนุนทางจิตใจ | ไม่จริง(1) | จริงบางครั้ง(2) | ค่อนข้างจริง(3) | จริงมาก(4) |
| --- | --- | --- | --- | --- | --- |
| 3.1 | หากฉันรู้สึกซึมเศร้า ฉันรู้ว่าจะโทรหาใครที่สามารถช่วยให้ฉันรู้สึกดีขึ้น |  |  |  |  |
| 3.2 | คนรอบข้างมักช่วยทำให้ฉันรู้สึกได้ถึงความห่วงใย |  |  |  |  |
| 3.3 | ฉันรู้ว่าใครที่ฉันสามารถพึ่งได้เมื่อตกอยู่ในภาวะวิกฤต |  |  |  |  |
| 3.4 | เวลาฉันรู้สึกซึมเศร้า ฉันออกจากความรู้สึกนั้น และพูดคุยกับผู้อื่น |  |  |  |  |
| 3.5 | ฉันปรับทุกข์กับผู้อื่นเพื่อสร้างและสานต่อความสัมพันธ์ที่ใกล้ชิด |  |  |  |  |

| 4 | การหลีกหนี ปัญหา | ไม่จริง(1) | จริงบางครั้ง(2) | ค่อนข้างจริง(3) | จริงมาก(4) |
| --- | --- | --- | --- | --- | --- |
| 4.1 | เวลามีปัญหา ฉันชอบพักมันไว้ก่อน |  |  |  |  |
| 4.2 | เมื่อพบกับปัญหาที่ยากเกินไป บ่อยครั้งที่ฉันปล่อยมันไว้รอจน ฉันพร้อมแล้วจึงจัดการกับมัน |  |  |  |  |
| 4.3 | เวลามีปัญหา ฉันจะปล่อยให้ไฟลนก้นแล้วจึงจัดการกับมัน |  |  |  |  |

**ส่วนที่ 4 คำถามเกี่ยวกับความต้องการเพิ่มเติมและข้อเสนอแนะที่ท่านคิดว่าหากปรับแล้วจะช่วยลดภาวะ Burnout**

กรุณาให้คะแนน 1-10 โดย 1 คือลดให้เหลือน้อยที่สุด, 5 คือไม่ต้องเปลี่ยนแปลง และ 10 คือเพิ่มให้มากที่สุด

- - 1. จำนวนผู้ปฏิบัติงาน ____
    2. จำนวนผู้ป่วยตรวจต่อวัน ____
    3. ชั่วโมงการทำงานต่อวัน ____
    4. จำนวนเวร ____
    5. งานเอกสาร ____
    6. วันหยุด ____
    7. อำนาจการบริหารในหน่วยงาน ____
    8. ความสนับสนุนโครงการใหม่ๆและนวัตกรรมในหน่วยงาน ____
    9. รายได้ ____
    10. การฝึกอบรมแพทย์ ____
    11. การฝึกอบรมผู้ร่วมงาน ____
    12. การสนับสนุนจากผู้บังคับบัญชา ____
    13. อุปกรณ์หรือเครื่องมือต่างๆในสถานที่ทำงาน ____
    14. งบประมาณของหน่วยงาน ____
    15. สวัสดิการในที่ทำงาน ____
    16. ความสัมพันธ์อันดีระหว่างผู้ร่วมงาน ____
    17. ความสนิทสนมระหว่างจิตแพทย์ด้วยกัน ____
    18. ความสัมพันธ์กับผู้บังคับบัญชา ____
    19. งานบริหาร ____
    20. บทบาทในฐานะแพทย์ทั่วไป เช่น อยู่เวรห้องฉุกเฉิน ตรวจผู้ป่วยทั่วไป ____
    21. การมีส่วนร่วมในการเปลี่ยนแปลงขององค์กร ____

ข้อเสนอแนะอื่นๆ________________________
