# Supplementary material for: Thai psychiatrists and burnout: A national survey
Source: PLoS One. 2020 Apr 21;15(4):e0230204. doi: 10.1371/journal.pone.0230204 (PMC7173626; doi:10.1371/journal.pone.0230204)
Supplement: S2 Appendix — (DOCX) [file pone.0230204.s002.docx]

**Research questionnaire**

Thai psychiatrists and burnout: a national survey

**Part 1** Sociodemographic data and job satisfaction questionnaire

1. Age

(Please select from 22-100)

1. Sex

🞎 Female 🞎 Male

1. Position

🞎 General psychiatrist 🞎 Child and adolescent psychiatrist 🞎Psychiatry resident

1. Experience as a psychiatrist (years)

(Please select from 1-70)

1. Marital status

🞎 Single 🞎 Married 🞎 Widow/divorced

1. Number of children

🞎 None 🞎 1 🞎 >1

1. Workplace (You can select more than 1 option)

🞎 Medical school/Teaching hospital 🞎 Psychiatric hospital

🞎 Regional hospital 🞎 General hospital

🞎 Community hospital 🞎 Private hospital/clinic

1. Which health district are you working in?

(Please select from district number 1-13, or 14 if not applicable, or 15 if do not know)

1. Number of patients per day (person)

(Enter a number :___)

1. Working hours per week

🞎 <40 🞎 40-50 🞎 >50

1. Number of shifts per month (including psychiatric shifts and other shifts)

(Please select from 1-31)

1. How satisfied were you with your current income?

Please rate from 0-10 (0 = least satisfied, 10 = extremely satisfied)

1. How satisfied were you with your current job?

Please rate from 0-10 (0 = least satisfied, 10 = extremely satisfied)

1. Days off per month

🞎 0-2 🞎 3-5 🞎 6-8 🞎 8-10 🞎 >10

1. Experienced with death of patients who committed suicide within :

🞎 None 🞎 ≤ 1 month

🞎 > 1 month - < 1 year 🞎 ≥ 1 year

1. How was the quality of support from :
   1. Chiefs of the psychiatric departments?

🞎 Good 🞎 Not good 🞎 N/A

- 1. Hospital administrative staff?

🞎 Good 🞎 Not good 🞎 N/A

- 1. Psychiatrist friends?

🞎 Good 🞎 Not good 🞎 N/A

- 1. Other colleagues?

🞎 Good 🞎 Not good 🞎 N/A

- 1. Doctors in other departments?

🞎 Good 🞎 Not good 🞎 N/A

- 1. Family?

🞎 Good 🞎 Not good 🞎 N/A

1. When you were faced with stress, who do you choose to consult or seek support? (You can select more than 1 option)

🞎 Yourself 🞎 Chiefs of the psychiatric department 🞎 Friends

🞎 Your partner 🞎 Your family 🞎 Social media 🞎 Other (please specify)

**Part 2 Thai version of the Maslach Burnout Inventory (MBI) questionnaire**

Summawart S. Burnout among the staff nurses in Ramathibodi Hospital. Bangkok: Mahidol University; 1989. Available from: <http://mulinet11.li.mahidol.ac.th/e-thesis/scan/18486.pdf>.

Maslach C, Jackson SE. The measurement of experienced burnout. J Organiz Behav. 1981;2(2):99-113. doi: 10.1002/job.4030020205.

<https://onlinelibrary.wiley.com/doi/abs/10.1002/job.4030020205>

**Part 3 Thai version of The Proactive Coping Inventory (PCI) questionnaire**

Tatha O, Laurujisawat P, Greenglass E. The validity and reliability of the Proactive Coping Inventory (PCI): Thai version. Chula Med J. 2013;57(6):765 - 78. Available from: <http://clmjournal.org/_fileupload/journal/32-8.pdf>.

Greenglass E, Schwarzer R, Jakubiec D, Fiksenbaum L, Taubert S. The Proactive Coping Inventory (PCI): a multidimensional research instrument. The 20^th^ International Conference of the Stress and Anxiety Research Society (STAR); July 12-14, 1999; Cracow, Poland 1999. Available from: <https://estherg.info.yorku.ca/files/2014/09/pci.pdf>.

### Part 4 Strategies that Thai psychiatrists believed could ameliorate their burnout

Please rate from 1-10 (1=should be reduced as much as possible, 5= no need to change, 10= should be increased as much as possible)

1. Number of staff members ____
2. The number of patients per day ____
3. The number of working hours per day ____
4. The number of shifts ____
5. The amount of paperwork ____
6. Days off ____
7. Administrative role in the department ____
8. Support for new projects and innovation in the department ____
9. Income ____
10. Training for psychiatrist ____
11. Training for team colleagues ____
12. Support from the head of the department ____
13. Workplace equipment ____
14. The departmental budget ____
15. Workplace welfare ____
16. Good relationship among team member ____
17. Good relationship among psychiatrists ____
18. Relationship with the head of the department ____
19. Administrative role in the hospital ____
20. The amount of general practice work, e.g., emergency room shift, general patient examination ____
21. Participation in changing of the organization ____

Further suggestions (please specify) ________________
