# Supplementary material for: Thai psychiatrists and burnout: A national survey
Source: PLoS One. 2020 Apr 21;15(4):e0230204. doi: 10.1371/journal.pone.0230204 (PMC7173626; doi:10.1371/journal.pone.0230204)
Supplement: Attachment — Submitted filename: Response to Reviewers.docx [file pone.0230204.s005.docx]

***Response to the Reviewer:***

1) **This is a second national burnout survey of Thai psychiatrists. The first national survey published in a Thai journal in 2011 by Lerthattasilp et al received a similarly low response rate of 27.8% (181/650), compared with the present study that has 27.15% (227/836). Despite the low response rate, the findings are still useful but characteristics of the responders should be compared with that of the non-responders if possible. Alternatively, the characteristics of all Thai psychiatrists is available in the national registry and should be presented alongside with the characteristics of the responders to this survey.**

We really appreciated the reviewer’s suggestion. Accordingly, we tried to find the characteristics of all Thai psychiatrists, but there were no official data available. We requested data from the registration office of the Psychiatric association of Thailand and categorized into the demographic data in Table 1, then compared with the characteristics of the respondents.

We also added the following sentences to the 8^th^ paragraph of the Discussion section.

“One limitation of our study was the small number of participants, which may limit the generalizability of our findings. We attempted to compare the characteristics of the respondents with the non-responders, but there are no official data of the general characteristics of Thai psychiatrists in the whole country. We did obtain data from the registration office of the Psychiatric Association of Thailand. (Table 1). The Psychiatric Association data suggest that 45% of Thai psychiatrists while 30% of our respondents were male. The Psychiatric Association reports that the mean years of experience of all psychiatrists is 17 years, while our respondents reported a mean of 8.9 years’ experience.”

2) **While the first national survey used only the Maslach Burnout Inventory (MBI), the present survey added the Proactive Coping Inventory (PCI) in order to investigate the relationship between coping style and burnout in Thai psychiatrists. The authors need to provide a rationale how these 4 of the 7 PCI scales were selected: proactive coping, strategic planning, emotional support seeking, and avoidance coping.**

Thank you for this comment. To clarify this issue, we modified the Materials and Methods section to emphasize the rationale how we selected these scales, as follows.

“The Thai version of the Proactive Coping Inventory (PCI) questionnaire [16, 17] is a multidimensional instrument that consists of 55 items divided into seven scales. The literature supports that burnout is associated with two major coping strategies: problem-focused coping and emotional-focused coping. Problem-focused coping includes proactive coping, directive coping, and plan resolution strategies. Emotional-focused coping covers positive reappraisal, seeking social support, and avoidance [18-20]. Because each PCI scale is independent from each other, the authors selected four scales to make the questionnaire more concise. Scale #1 is proactive coping defined as the combination of autonomous goal setting with self-regulatory goal attainment cognition and behavior. Scale #2 is strategic planning defined as the process of generating a goal-oriented schedule of action in which extensive tasks are broken down into more manageable tasks. Scale #3 is emotional support seeking defined as the regulation of temporary emotional distress by disclosing feelings to others, evoking empathy and seeking companionship from one’s social network. Last, Scale #4 is avoidance coping defined as the illusion of action in a demanding situation by delaying action. There was no cut-off point for each scale; a higher score indicated a greater degree of each coping mechanism. The Cronbach’s alpha coefficient of each PCI domain in the Thai version is between 0.70-0.81 [17].”

3) **The correlation between MBI and PCI subscales were assessed by using Pearson's correlation coefficients. As the MBI contains 22 items in 3 subscales whereas the PCI contains 55 items in 7 subscales (but 26 items in 4 subscales were selected in this study, normality of both variables had to be checked.**

Thank you for pointing this out. We agree with this comment. We checked both of the variables and the results showed that both of them were non-normality data. Therefore, we reanalyzed the data with the Spearman correlation and revised our manuscript. The new analysis showed the same significant findings.
